# Hepatic Stearoyl-CoA desaturase-1 deficiency-mediated activation of mTORC1- PGC-1α axis regulates ER stress during high-carbohydrate feeding

**DOI:** 10.1038/s41598-019-52339-7

**Published:** 2019-10-31

**Authors:** Ahmed Aljohani, Mohammad Imran Khan, Deeba N. Syed, Bonneville Abram, Sarah Lewis, Lucas O’ Neill, Hasan Mukhtar, James M. Ntambi

**Affiliations:** 1 School of Medicine and Public Health, Endocrinology and Reproductive Physiology Graduate Training Program, University of Wisconsin-Madison, Madison, WI 53706 USA; 2 King Saud bin Abdulaziz University for Health Sciences, National Guard Health Affairs, Riyadh, 11426 Saudi Arabia; 3 Department of Biochemistry, Faculty of Science, King Abdulaziz University, Jeddah, 80203 Saudi Arabia; 4 Department of Dermatology, School of Medicine and Public Health, University of Wisconsin-Madison, Madison, WI 53706 USA; 5 Department of Biochemistry, University of Wisconsin-Madison, Madison, WI 53706 USA; 6 Department of Nutritional Sciences, University of Wisconsin-Madison, Madison, WI 53706 USA

**Keywords:** Carbohydrates, Fatty acids

## Abstract

Stearoyl CoA desaturase 1 (SCD1) is a key enzyme in lipogenesis as it catalyzes the synthesis of monounsaturated fatty acids (MUFAs), mainly oleate (18:1n9) and palmitoleate (16:1n7) from saturated fatty acids (SFA), stearate (18:0) and palmitate (16:0), respectively. Studies on SCD1 deficiency in mouse models demonstrated beneficial metabolic phenotypes such as reduced adiposity and improved glucose tolerance. Even though, SCD1 represents a potential target to resolve obesity related metabolic diseases; SCD1 deficiency causes endoplasmic reticulum (ER) stress and activates unfolded protein response (UPR). The induction of ER stress in response to SCD1 deficiency is governed by the cofactor, PGC-1α. However, the mechanism by which SCD1 deficiency increases PGC-1α and subsequently induces ER stress still remains elusive. The present study demonstrates that despite reduced lipogenesis, liver specific SCD1 deficiency activates the mechanistic target of rapamycin complex 1 (mTORC1) along with induction of PGC-1α and ER stress. Further, mTORC1 inhibition attenuates SCD1 deficiency-mediated induction of both PGC-1α and ER stress. Similar observations were seen by restoring endogenously synthesized oleate, but not palmitoleate, suggesting a clear mTORC1-mediated regulation of ER stress during SCD1 deficiency. Overall, our results suggest a model whereby maintaining adequate levels of hepatic oleate is required to suppress mTORC1-mediated ER stress. In addition, the activation of mTORC1 by SCD1 deficiency reveals an important function of fatty acids in regulating different cellular processes through mTORC1 signaling.

## Introduction

Stearoyl CoA desaturase 1 (SCD1) is a key enzyme in lipogenesis catalyzing the rate limiting step in the synthesis of monounsaturated fatty acids (MUFA). MUFA are preferentially incorporated into different complex lipid species fulfilling different cellular signaling, structural and energy storage functions. Previous studies showed positive correlation between MUFA levels and human obesity^1,2^. Also, MUFA plasma levels were elevated in hyperlipidemic conditions, implicating an important role of MUFA in obesity related chronic diseases^3^. Consistently, many research studies showed that targeting SCD1 activity is sufficient to attenuate obesity related chronic diseases, indicating that modulation of SCD1 activity may confer many beneficial health outcomes.

The mechanistic target of rapamycin (mTOR) is an evolutionarily conserved serine/threonine protein kinase that integrates diverse signals to modulate anabolic and catabolic cellular processes. mTOR is the catalytic subunit of two functionally distinct complexes, mTORC1 and mTORC2, which regulate diverse cellular functions^4^. Of the two complexes, mTORC1 has been shown to regulate lipogenesis through enhancing sterol regulatory element binding protein-1c (SREBP)-1c processing and nuclear translocation which subsequently increased the expression of lipogenic genes, including fatty acid synthase (FAS), Acetyl-CoA carboxylase (ACC) and SCD1^4,5^. Moreover, treating with mTORC1 inhibitor, rapamycin, suppressed fasting-refeeding mediated induction of lipogenesis^6^. Studies of mTORC1 regulation illustrated that mTORC1 acts as a sensing kinase activated by nutrients including glucose and amino acids or growth hormones such as insulin to regulate downstream functions^4^. Similarly, different fatty acids, as nutrients, have been shown to modulate mTORC1 activity in different cell types^7–9^. However, the differential effects of MUFA on mTORC1 activity *in vivo* have not been fully elucidated.

Metabolic profiling of SCD1 demonstrated that global deletion of SCD1 leads to profound protection against diet-induced adiposity and liver steatosis. Likewise, hepatic SCD1 deficiency was sufficient to reduce high carbohydrate diet (HCD) induced adiposity with a significant reduction of hepatic lipogenesis and improved glucose tolerance^10^. Despite preferred metabolic phenotypes, SCD1 deficiency was associated with induction of ER stress and UPR activation. We recently showed that induced expression of ER stress genes in response to SCD1 deficiency is mediated through peroxisome proliferator-activated receptor gamma coactivator-1 alpha (PGC-1α)^11^. However, the precise mechanism by which SCD1 deficiency upregulates PGC-1α and subsequently ER stress is not fully understood.

In this study, we sought to determine the mechanism by which SCD1 deficiency induces ER stress. HCD feeding study revealed that SCD1 deficiency activates mTORC1 signaling pathway and uncouples active mTORC1 mediated lipogenesis. In response to SCD1 deficiency, active mTORC1 contributes to the induction of PGC1α mediated ER stress. To provide the proof of principle, we used two transgenic mouse models that overexpress either human SCD5 or mouse SCD3 in the liver of SCD1 global knockout mice to delineate the differential effects of endogenously synthesized hepatic oleate or palmitoleate, respectively, on mTORC1 activity. We found that restoring hepatic oleate levels, but not palmitoleate, inactivates mTORC1, reduces the expression of PGC-1α and resolves ER stress. Oleate mediated suppression of mTORC1 was also observed in the liver of LKO mice fed triolein, but not tristearin, supplemented HCD. These findings indicate a pivotal role of hepatic oleate to suppress mTORC1 signaling and thereby mTORC1 mediated ER stress. Also, this study provides valuable insight into the involvement of fatty acids in modulating cellular responses through mTORC1.

## Results

### SCD 1 deficiency activates mTORC1

**Figure 1:**
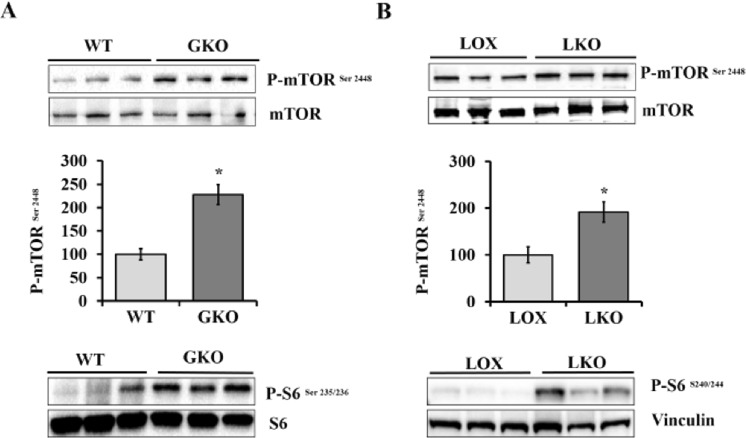
SCD1 deficiency activates mTORC1 signaling. (**A**) 10 weeks old male WT and SCD1 GKO mice were fed HCD for 10 days. Western blot with the indicated antibodies performed on liver samples. (**B**) 10 weeks old male LOX and LKO mice were fed HCD for 10 days. Western blot with the indicated antibodies performed on liver samples. The relative phosphorylation levels of mTOR-Ser 2448 was quantified and normalized to total mTOR using densitometry. Values are mean ± SEM (n = 3/group), *P < 0.05 vs LOX counterparts by student’s two-tailed *t* test.

Our previous reports of reduced hepatic lipogenesis in response to SCD1 deficiency prompted us to study the signaling pathways that govern the expression of lipogenic genes^10,12^. mTORC1 is one of the signaling pathways that have been shown to regulate the expression of lipogenic genes, including SCD1, mainly through promoting SREBP1c maturation and nuclear translocation^5,13^. To investigate the effect of SCD1 deficiency on mTORC1 signaling pathway, we used SCD1 global knockout (GKO) and control wild type (WT) mice. All mice were fed a high carbohydrate diet (HCD), which has low fat content, to potently induce lipogenesis and to assess the role of endogenous MUFAs in regulating mTORC1 signaling pathway^10^. Mice were fed HCD diet for 10 days and liver tissues were collected at the end of the feeding period. Using immunoblot analysis, we determined the phosphorylation status of mTOR in liver tissue. mTOR Ser2448 phosphorylation was significantly increased in the liver of SCD1 GKO mice compared with WT mice, suggesting a clear mTORC1 activation (Fig. 1A). To further assess mTORC1 signaling pathway activity, we determined the phosphorylation levels of ribosomal S6 protein, a downstream target of mTORC1 signaling pathway. The liver of SCD1 GKO mice showed increased ribosomal S6 protein phosphorylation when compared to control mice, confirming mTORC1 activation in response to SCD1 deficiency (Fig. 1A). Next, to evaluate the specific role of hepatic SCD1 in regulation of mTORC1 activity, we used LKO mice lacking SCD1 exclusively in the liver and their floxed littermates (LOX) mice with intact SCD1 expression. Like SCD1 GKO mice, the liver of LKO mice fed HCD exhibited increased mTOR phosphorylation compared to LOX control mice (Fig. 1B). Higher ribosomal S6 protein phosphorylation was also observed in the liver of LKO mice (Figs 1B and S1). Thus, loss of global and hepatic SCD1 results in mTORC1 activation in response to HCD fed mice.

### SCD1 deficiency uncouples active mTORC1-regulated lipogenesis

**Figure 2:**
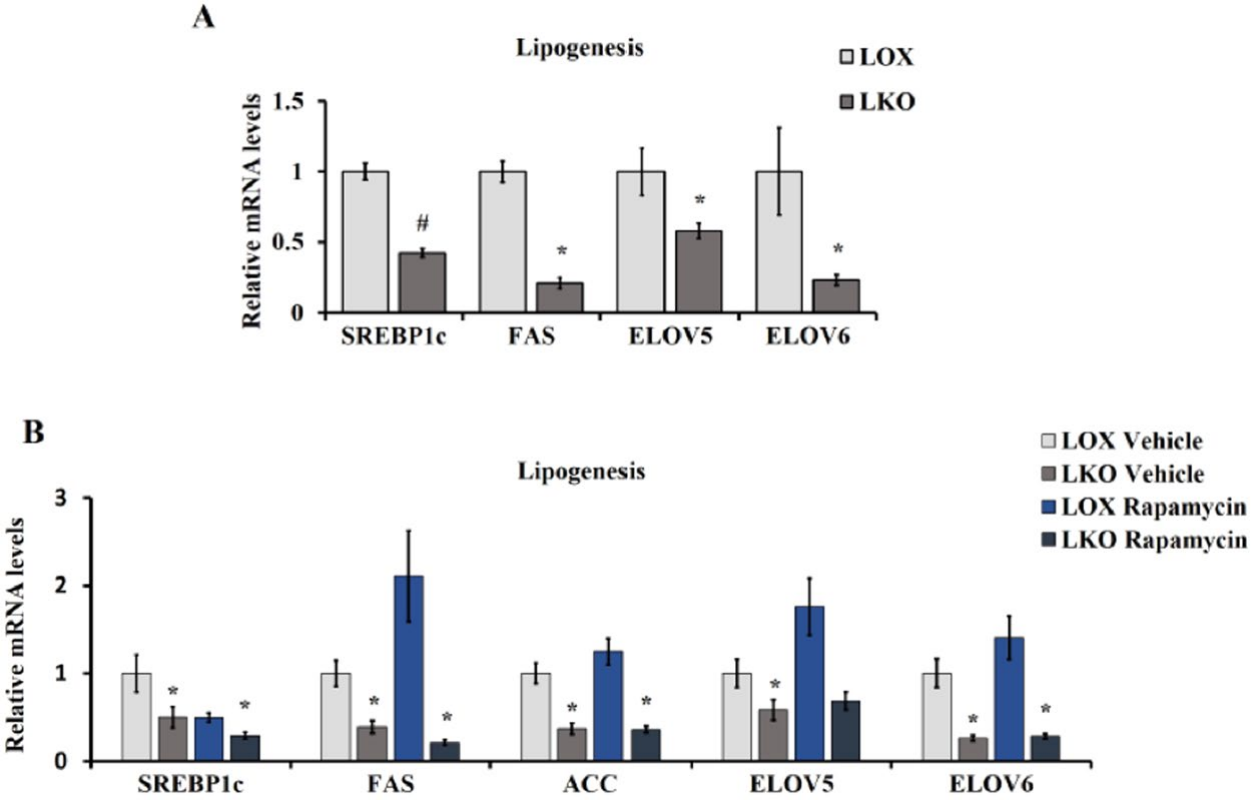
SCD1 deficiency uncouples active mTORC1-regulated lipogenesis. (**A**) 10–12 weeks old male LOX and LKO mice were fed HCD for 10 days. (**B**) 10 weeks old male LOX and LKO mice were fed HCD and treated with rapamycin 2 mg/Kg/day body weight daily for 10 days. Liver gene expression analysis for the indicated lipogenic genes was carried out using real time PCR. Values are mean ± SEM (n = 4–6/group), *P < 0.05 vs LOX counterparts by student’s two-tailed *t* test.

The mRNA expression of lipogenic genes were measured in the liver of LKO and LOX mice fed HCD. Despite a clear mTORC1 activation in response to SCD1 deficiency, LKO mice showed lower expression of lipogenic genes namely SREBP1c, FAS, fatty acid elongase 5 (Elov5) and fatty acid elongase 6 (Elov6) compared with control mice, indicating reduced lipogenesis (Fig. 2A). These results are consistent with our previous work showing reduced rate of de novo lipogenesis in the liver of LKO mice^10^. Studies on Tuberous sclerosis 1 (TSC1), a negative regulator of mTORC1, deficient models demonstrated that mTORC1 hyperactivation decreases lipogenesis through a feedback inhibition of insulin signaling^13,14^. To examine this decrease in lipogenesis in response to SCD1 deficiency-mediated mTORC1 activation, LKO mice were fed HCD and treated with 2 mg/Kg/day rapamycin, an allosteric inhibitor of mTORC1, for 10 days. Rapamycin treated LKO mice displayed no significant alteration in the expression of lipogenic genes compared with vehicle treated LKO mice (Fig. 2B). These results indicate that SCD1 or its products are indispensable for lipogenesis and active mTORC1 has no effect on lipogenesis in LKO mice.

### SCD1 deficiency enhances PGC-1α expression through mTORC1

**Figure 3:**
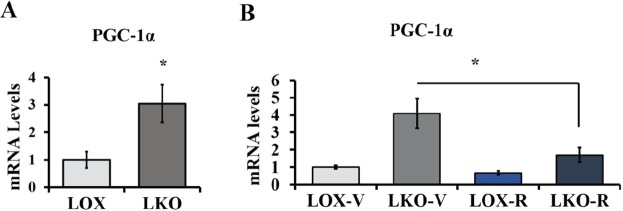
SCD1 deficiency enhances PGC-1α expression through mTORC1 activation. (**A**) 10–12 weeks old male LOX and LKO mice were fed HCD for 10 days. (**B**) 10 weeks old male LOX and LKO mice were fed a HCD and treated with rapamycin 2 mg/Kg body weight daily for 10 days. Liver PGC-1a gene expression was analyzed as indicated using real time PCR. Values are mean ± SEM (n = 4–6/group), *P < 0.05 vs LOX counterparts and ^#^P < 0.05 vs vehicle treated LKO by student’s two-tailed *t* test.

Consistent with our previous studies, SCD1 deficiency increases PGC-1α expression in the liver (Fig. 3A)^10^. Active mTORC1 has been previously shown to increase the expression of PGC-1α in the liver of TSC1 liver specific knockout mice^15^. Therefore, to determine whether active mTORC1 is involved in the upregulation of PGC-1α, LKO mice fed HCD were administered rapamycin and PGC1α mRNA expression levels were evaluated. Interestingly, mTORC1 inhibition led to a significant reduction of PGC-1α expression compared to its expression in the liver of vehicle treated LKO mice (Fig. 3B). These results indicate that increased levels of SFA in response to SCD1 deficiency may increase PGC-1α through enhancing mTORC1 activity.

### mTORC1 contributes to ER stress via PGC-1α in SCD1 deficient conditions

**Figure 4:**
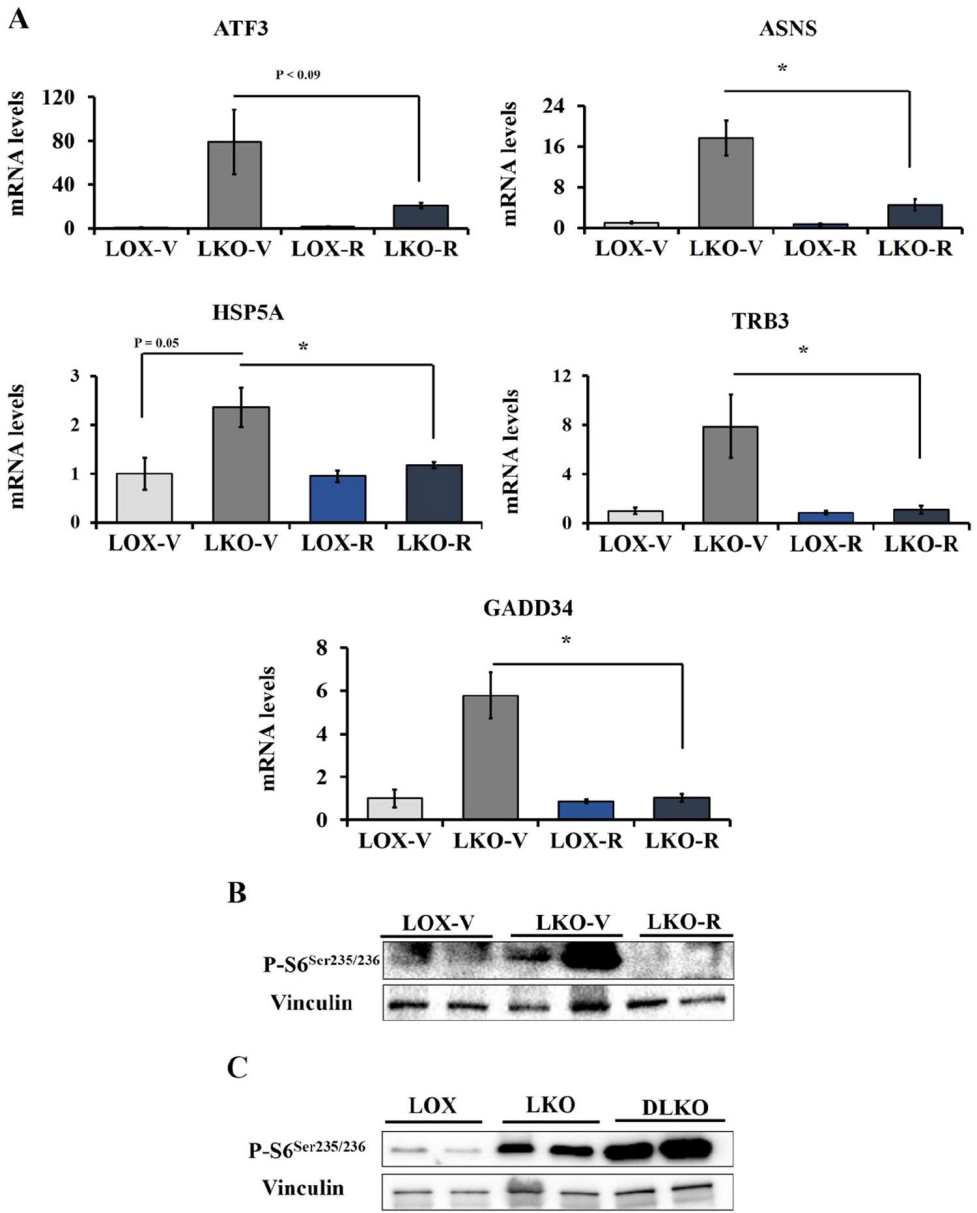
mTORC1 contributes to SCD1 deficiency mediated induction of ER stress genes through PGC-1α. (**A**) 10–12 weeks old male LOX and LKO mice were fed HCD and treated with rapamycin 2 mg/Kg body weight daily for 10 days. Liver gene expression analysis for the indicated ER stress genes was carried out using Qpcr. (**B**) Western blot with the indicated antibodies performed on liver samples. (**C**) 10 weeks old male LOX, LKO and DLKO mice were fed HCD. Western blot with the indicated antibodies performed on liver samples. Values are mean ± SEM (n = 4–6/group), *P < 0.05 vs LOX counterparts and # P < 0.05 vs vehicle treated LKO by student’s two-tailed *t* test.

SCD1 deficiency leads to number of favorable metabolic phenotypes including reduced body weight and improved glucose tolerance in diet-induced obesity mouse models. However, despite positive outcomes, reduced SCD1 activity is associated with induced ER stress. LKO mice demonstrated increased expression of ER stress genes including activating transcription factor 3 (ATF3), growth arrest and DNA damage-inducible protein (GADD34), asparagine synthetase (ASNS), and heat shock protein 5 (HSPA5)^11^. To investigate the role of mTORC1 in SCD1 deficiency-induced ER stress, we assessed the expression of ER stress genes in the liver of rapamycin treated LKO mice. mTORC1 inhibition exhibited a dramatic reduction of ER stress genes in LKO mice compared with vehicle treated LKO mice (Fig. 4A). mTORC1 inhibition in the liver of LKO mice was assessed using ribosomal S6 protein phosphorylation (Fig. 4B). Both ATF3 and GADD34 which are down stream of the eIF2a arm of ER stress were significantly reduced by rapamycin treatment suggesting that mTORC1 induces ER stress in response to reduced SCD1 activity. We have recently shown that the upregulation of ER stress genes in response to SCD1 deficiency were normalized in SCD1/PGC-1α double knockout (DLKO) mice which indicates that SCD1 deficiency increases ER stress through PGC-1α^11^. Since rapamycin treatment reduced PGC-1α and ER stress genes in LKO mice, we wanted to determine the status of mTORC1 in the liver of DLKO mice. Interestingly, similar to LKO mice, the liver of DLKO mice showed increased S6 protein phosphorylation compared with LOX mice (Fig. 4C). Sustained activation of mTORC1 in DLKO mice indicates that PGC-1α deletion abolishes mTORC1 effects on ER stress. Also, reduced ER stress genes in response to rapamycin treatment suggests that active mTORC1 contributes to SCD1 deficiency-mediated ER stress through promoting PGC-1α expression. Furthermore, we examined the effect of SCD1 deficiency on Akt phosphorylation (Fig. S2). The liver of LKO mice showed increased Akt phosphorylation which was sustained in the liver of DLKO mice. These results may indicate promoted insulin signaling upon SCD1 deficiency.

### Oleate, but not palmitoleate, deactivates mTORC1 signaling and resolves ER stress

**Figure 5:**
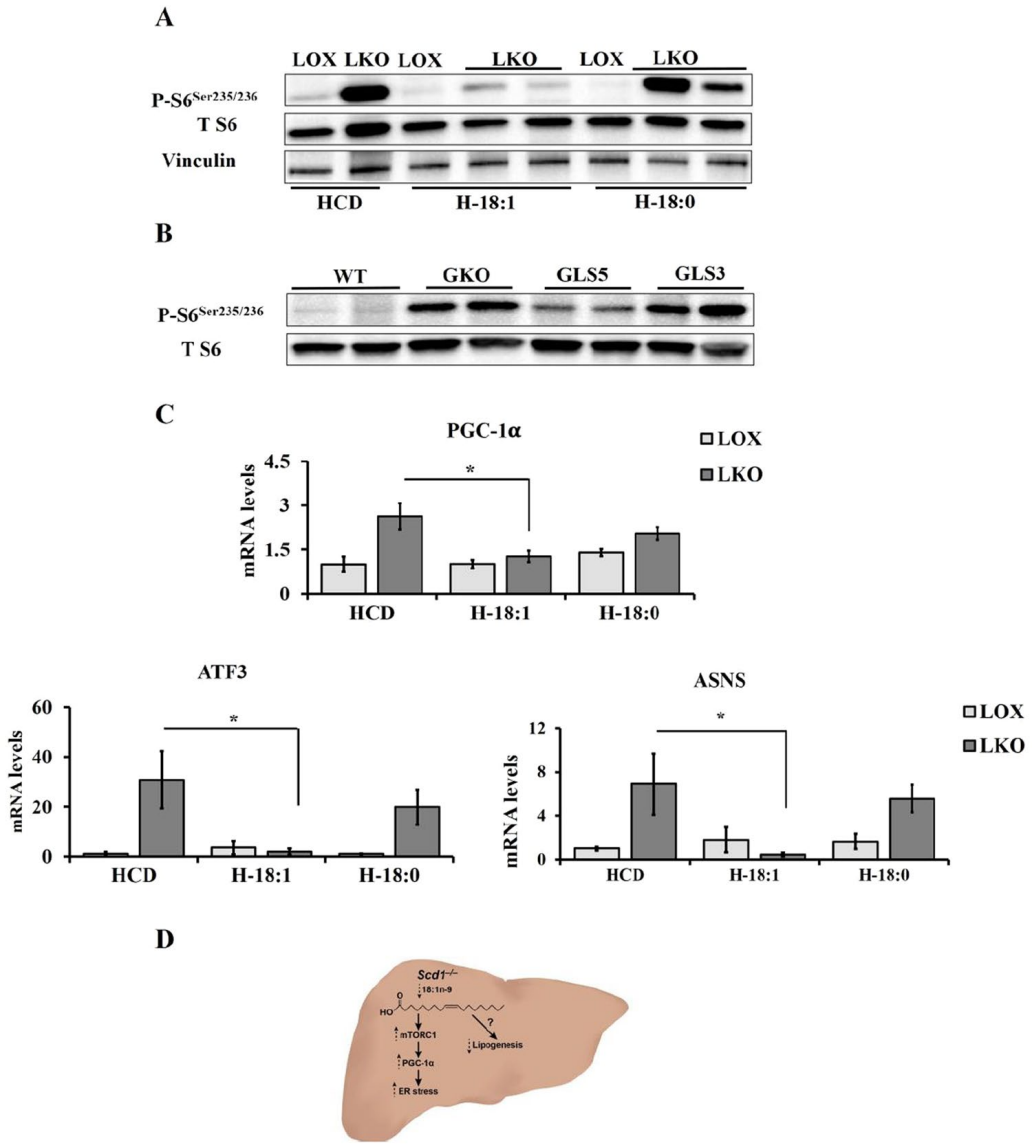
Oleate, but not palmitoleate, deactivates mTORC1 and resolves ER stress *in vivo*. (**A**) 10–12 weeks old male WT, SCD1 GKO, GLS5, and GLS3 were fed HCD for 10 days. Western blot with the indicated antibodies performed on liver samples. (**B**,**C**) 12 weeks old male LOX and LKO mice were fed HCD, H-18:1, or H-18:0 for 10 days. Western blot analysis with the indicated antibodies and liver gene expression analysis of ER stress genes using Qpcr. Values are mean ± SEM (n = 3–6/group), *P < 0.05 vs LOX counterparts and #P < 0.05 vs vehicle treated LKO by student’s two-tailed *t* test. (**D**) Proposed mechanism: Hepatic SCD1 deficiency increases palmitate which subsequently promotes mTORC1-mediated ER stress through PGC-1α.

We have previously shown that restoring oleate levels through endogenous or exogenous sources reduces ER stress in SCD1 GKO or LKO mice, respectively^10^. Moreover, since rapamycin reduces ER stress, we hypothesized that oleate feeding might deactivate mTORC1 and subsequently reduce ER stress. To examine this hypothesis, LOX and LKO mice were fed either triolein supplemented HCD, as a source of oleate, or tristearin supplemented HCD, as a control to assess the effect of further accumulation of SCD1 substrate. The ribosomal S6 protein phosphorylation levels were evaluated in the liver of these mice. Triolein supplemented HCD decreased S6 phosphorylation whereas tristearin supplemented diet failed to do so in the liver of LKO mice (Fig. 5A). To further explore the effect of endogenously synthesized MUFA, oleate and palmitoleate, on mTORC1 activation, we used two liver transgenic mouse models that overexpress either human SCD5 (GLS5) or mouse SCD3 (GLS3). These enzymes preferentially catalyze oleate and palmitoleate synthesis, respectively, from their precursor SFA^11,16^. The liver of GLS5 mice showed lower ribosomal S6 protein phosphorylation while the phosphorylation level remained elevated in GLS3 mice similar to GKO mice (Fig. 5B). In addition, we analyzed the expression of ER stress genes in the liver of LKO mice fed triolein or tristearin supplemented HCD. We found that triolein, but not tristearin, supplemented HCD reduces the expression of PGC-1α and ER stress genes in the liver of LKO mice (Fig. 5C). These findings suggest that oleate is required for mTORC1 mediated induction of lipogenesis. Reduced oleate levels may direct mTORC1 activities towards PGC-1α mediated ER stress in the liver of SCD1 deficient mice (Fig. 5D).

## Discussion

In the present study, we determined the effect of SCD1 deficiency and investigated the differential effects of endogenous MUFA supplementation on mTORC1 signaling pathway, which is a critical regulator of hepatic *de novo* lipogenesis. We demonstrate for the first time using *in vivo* mouse models that SCD1 deficiency in the liver leads to mTORC1 activation and induction of PGC-1α and ER stress, despite reduced lipogenesis. Next, we found that mTORC1 inhibition attenuated SCD1 deficiency-induced PGC-1α and ER stress. Moreover, restoring endogenously synthesized oleate, but not palmitoleate, suppresses mTORC1 activation in the liver of SCD1 GKO mice. These results reveal a model in which active mTORC1 is not sufficient to induce lipogenesis despite enhanced Akt phosphorylation (Fig. S2) but instead contributes to ER stress in response to SCD1 deficiency, suggesting a novel facet of mTOR signaling in hepatic *de novo* lipogenesis.

It is well established that mTORC1 signaling pathway integrates nutritional and growth factors signals dictating cellular responses through controlling different signaling and metabolic pathways. In response to insulin or glucose, active mTORC1 increases the expression of lipogenic genes including SCD1 and rapamycin treatment suppresses insulin or glucose mediated lipogenesis^17^. Interestingly, despite reduced lipogenesis, the liver of SCD1 GKO and LKO mice showed higher phosphorylation of mTOR and ribosomal S6 protein suggesting mTORC1 activation. mTORC1 activation in response to SCD1 deficiency is consistent with previous studies that showed saturated fatty acids involvement in the activation of mTORC1 (7). Also, the failure of mTORC1 to induce lipogenesis in the case of SCD1 deficiency indicates that MUFA are indispensable for lipogenesis.

SCD1 deficiency increases the ratio of SFA to MUFA and this change in fatty acids composition suggests that SCD1 deficiency activates mTORC1 through increased SCD1 substrate, palmitate or stearate. In L6 myotubes, palmitate, a saturated fatty acid, enhances insulin-mediated activation of mTORC1. Similarly, palmitate activates mTORC1 through promoting mTORC1 translocation onto lysosomal membrane^7–9^. Previous data in combination with the findings presented in this study suggest that increased palmitate or stearate levels activate mTORC1 in response to SCD1 deficiency. mTORC1 activation in response to SCD1 deficiency is more likely to be a result of increased palmitate than stearate as tristearin feeding to LKO mice did not induce further activation of mTORC1 (Fig. 5A). Consistent with this, tristearin supplemented diet did not show differences in ribosomal S6 protein phosphorylation levels in the liver of control LOX mice. SCD1 deficiency-enhanced mTORC1 activation is in line with previously reported work indicating that SCD1 is required for autophagy, a cellular process inhibited by active mTORC1^18^. However, whether both palmitate and stearate activate mTOR needs further study.

SCD1 deficiency has been previously shown to activate AMPK in the liver of SCD1 GKO mice, whereas the liver of LKO mice showed no change in AMPK phosphorylation^10,19^. However, the present work suggests that mTORC1 is activated in the liver of both SCD1 GKO and LKO mice. Despite feeding HCD, SCD1 GKO mice become very hypoglycemic after fasting four hours before being euthanized for analysis. This may indicate that AMPK was activated during the fasting period but was not sufficient to inhibit the mTORC1 activation. It might be also possible that elevated palmitate ratio upon SCD1 deficiency further enhances mTORC1 and AMPK activation independent of each other. However, this assumption needs to be explored in future studies.

Human SCD5 and mouse SCD3 isoforms preferentially catalyze the synthesis of oleate and palmitoleate, respectively^16^. The generation of GLS5 and GLS3 transgenic mice enabled us to determine the effect of endogenously synthesized MUFAs on mTORC1 activation and ER stress. Similar to our chemical based inhibition of mTORC1 we found that reduction in mTORC1 activation in response to elevated endogenous oleate, further indicates that restoring oleate, but not palmitoleate, suppresses palmitate-mediated mTORC1 activation. Similarly, oleate has been previously shown to abolish palmitate-induced activation of mTORC1 in podocytes^8^. However, on the other hand, these results are opposite to that of oleate treated human liver cancer cell line (HepG2) which demonstrated enhanced proliferation through promoting phospholipase D and mTORC1 activation^20^. Although further studies are required to address such discrepancies, our *in vivo* study clearly showed no significant difference in S6 protein phosphorylation between triolein supplemented HCD and HCD fed LOX mice. Therefore, it might be possible that oleate confers different effects *in vitro* compared with *in vivo* conditions.

UPR is a cytoprotective signaling pathway triggered in response to accumulating unfolded proteins or nutrient fluctuations^21^. In turn, UPR activation helps cells adapt to stress or mediates apoptosis in cases of persistent stress. Recently, we showed that the imbalance between MUFA and SFA induced by SCD1 deficiency causes ER stress and UPR activation which was resolved after restoring oleate levels^11^. Also, upregulated PGC-1α in response to SCD1 deficiency mediates UPR activation as PGC-1α deletion was sufficient to reduce the expression of ER stress genes. Here, our study provides a clear link between active mTORC1 and increased ER stress in the liver of SCD1 deficient mice. Previously, in the tuberous sclerosis complex (TSC2) deficient animals and mouse embryonic fibroblasts (MEFs), dysregulated mTORC1 activity enhanced ER stress through UPR activation^14^. Similarly, mTORC1 activation in podocytes preceded UPR activation responsible for proteinuria and treating with everolimus, mTORC1 inhibitor, reduced UPR activity^22^. The mechanism by which mTORC1 increases the expression of ER stress genes is not completely understood. However, it is well accepted that active mTORC1-enhanced protein synthesis is the driver force for UPR activation and transcriptional mediators are not intensively investigated. Here, we show that mTORC1 inhibition in LKO mice exhibits significant downregulation of PGC-1α compared with vehicle treated LKO mice, suggesting that SFA may promote PGC-1α through enhancing mTORC1 activity. mTORC1 has been previously shown to modulate the expression of an array of genes involved in mitochondrial functions through PGC-1α and the transcription factor ying –yang 1 (YY1)^23^. Likewise, our work here suggests that PGC-1α works downstream of mTORC1 to promote the expression of ER stress genes in response to SCD1 deficiency (Fig. 5D). Taking all these together, mTORC1 activation in response to SCD1 deficiency may provide an insight on the mechanism by which saturated fat increases ER stress through PGC-1α. Furthermore, this study also provides a novel insight on how oleate levels may direct mTORC1 activity either towards lipogenesis or other pathways including ER stress.

## Methods

### Animal and diets

All animal studies were approved by and carried out in accordance to the Institutional Animal Care and Use Committee guidelines, University of Wisconsin-Madison, protocol # A005125. Mice were maintained at the University of Wisconsin-Madison animal care facility on regular 12 hours light/dark cycles with free access to food and water. They were fed a standard rodent chow diet (Purina 5008) unless otherwise stated. All studies were carried out using 8 to 12 weeks old mice. The process of generating SCD1 global knockout (GKO) mice, SCD1 ^l^°^x/l^°^x^ (LOX) control mice and SCD1 ^l^°^x/lox^; Albumin Cre/+ tissue specific liver knockout (LKO) mice and SCD1/PGC-1α double knockout (DLKO) mice was previously described^10,11^. All experiments were carried out using C57BL/6 mice background. For studies on endogenously synthesized MUFA, we used two transgenic mouse models that exclusively express either human SCD5 (GLS5) or mouse SCD3 (GLS3) in the liver of SCD1 global knockout background, as previously prescribed^11^. For some experiments, mice were fed high carbohydrate diet (HCD), which has low fat content, for a period of 10 days. Triolein or tristearin supplemented HCDs were prepared by supplementing the fat-free basal mix (TD150776.PWD; Harlan Teklad) with 15% by weight of tristearin (T5016; Sigma) or triolein (99% purity, T7140; Sigma). In other studies, mice were treated intraperitoneally with 2 mg/kg/day rapamycin (from LC laboratories) dissolved in saline and 2% ethanol for 10 days^24^. All mice from each group were fasted for 4 hours before being euthanized with isoflurane overdose. Collected tissues were snap frozen in liquid nitrogen and stored at −80 °C for future analysis.

### Real-time quantitative PCR analysis

Total RNA isolation was performed using Tri reagent (Molecular Research Center) and subsequently treated with Turbo DNase (Ambion). Isolated RNA was reverse transcribed with a high capacity cDNA reverse transcription kit (applied Biosystems). Real-time quantitative PCR analysis was performed using Syber Green Master Mix (Applied Biosystems) and an ABI 7500 instrument (Applied Biosystem). Relative mRNA expression levels were determined using the comparative CT method. Used primer sequences are available upon request.

### Immunoblot analysis

An aliquot of frozen liver samples was homogenized by using (hand held homogenizer) in ice-cold lysis buffer (50 mM Tris-HCL, 150 mNaCl, 1 mM EGTA, 1 mM EDTA, 20 mM Na3 VO4, 0.5% NP-40, 1% Triton X-100, 1 mM PMSF) with protease inhibitor (Protease inhibitor Cocktail Set III, Calbiochem, La Jolla, CA). After homogenization, samples were passed through needle to ensure complete cell lysis and then centrifuged 14,000 rpm for 20 min at 4 °C. The supernatant was immediately used or stored at −80 °C. For immunoblot analysis, 30 µg protein was resolved on 8–12% polyacrylamide gels and transferred to a nitrocellulose membrane. Membranes were incubated with primary antibody at 4 °C for overnight followed with incubation with anti-rabbit or anti-mouse secondary antibody horseradish peroxidase conjugate. All antibodies were purchased from Cell Signaling Technology, Inc. (Danvers, MA, USA) and Abcam (Cambridge, MA). Blots were developed by using chemiluminescence and autoradiography was done by using Bio-Rad Gel Doc (Bio-Rad laboratories In., Hercules, CA)

### Statistical analyses

Results are presented as mean ± SEM. Pair-wise comparisons were performed using an unpaired, two-tailed student’s *t* test. Results with a *P* value < 0.05 were considered statistically significant.

## Supplementary information

Supplementary figures 1 and 2

## Supplementary information

is available at this paper at 10.1038/s41598-019-52339-7.
