# Supplementary material for: Hepatic Stearoyl-CoA desaturase-1 deficiency-mediated activation of mTORC1- PGC-1α axis regulates ER stress during high-carbohydrate feeding
Source: Sci Rep. 2019 Oct 31;9:15761. doi: 10.1038/s41598-019-52339-7 (PMC6823547; doi:10.1038/s41598-019-52339-7)
Supplement: Supplementary file 1 — Supplementary figures 1 and 2 [file 41598_2019_52339_MOESM1_ESM.pdf]

Hepatic Stearoyl-CoA desaturase-1 deficiency-mediated activation of mTORC1- PGC-1α axis regulates ER stress during high-carbohydrate feeding

Ahmed AlJohani, Mohammad Imran Khan, Deeba N. Syed, Bonneville Abram, Sarah Lewis, Lucas O'neill, Hasan Mukhtar, James M. Ntambi

S1

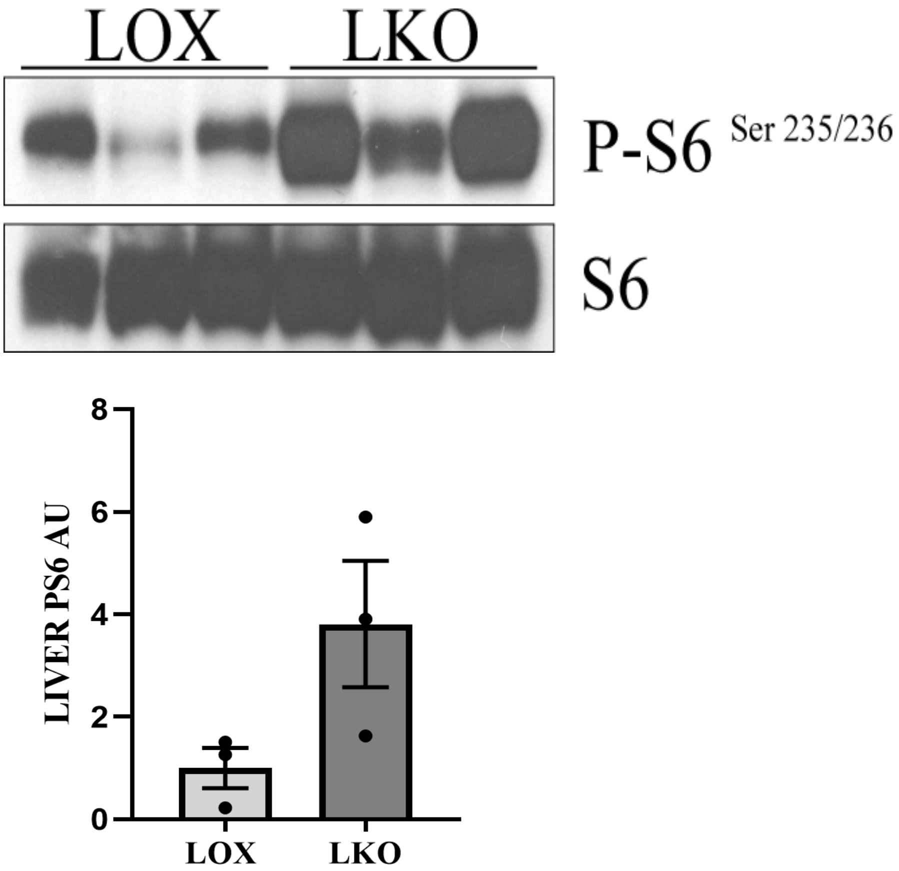
LOX
LKO
P-S6 Ser 235/236
S6
8
6
4
2
0
LIVER PS6 AU
LOX
LKO

S2

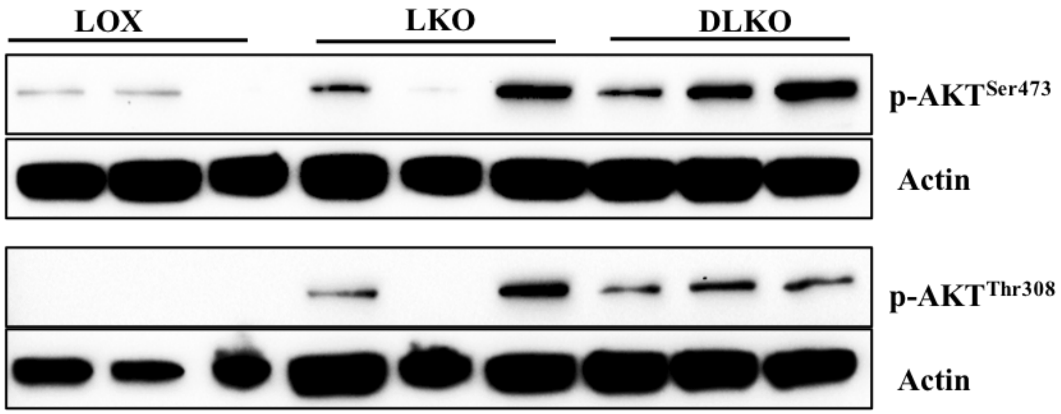
LOX
LKO
DLKO
p-AKTSer473
Actin
p-AKTThr308
Actin
